# Case of Extra-Uterine Pregnancy

**Published:** 1901-03

**Authors:** Chas. Wilson

**Affiliations:** Interne


### Clinical Report.

### CASE OF EXTRA-UTERINE PREGNANCY.

By Dr. L. G. HANLEY,
Gynecologist, Buffalo Hospital of the Sisters of Charity.

Reported by CHAS. WILSON, M. D., Interne.

MRS. J., age 32, admitted to hospital January 15, 1901. Number of children, two; abortions, 3; menses have been regular till September 2, 1900, when they ceased. Six weeks from date of admission flowed for twenty-one days; has had abdominal pains and was confined to her bed for four weeks; complains of most pain in left side of abdomen, low down, radiating over region of bladder; frequent desire to urinate and act slow. She has had at different intervals during the past three months several fainting spells preceded by pain, and been obliged to summon her physician, who she says placed her in bed and ordered stimulants. Has been badly bloated at times; and gives a history of a brownish discharge from the vagina.

Examination revealed a mass in left ovarian region size of child's head; uterus pushed to right side. Diagnosis: ruptured hematoma. The usual preparation given and abdomen opened through median line. The peritoneum was adhered to the mass and showed the effects of inflammatory action; there had been rupture not only between folds of broad ligament but into abdominal cavity as well; adhesions broken up, and specimen enucleated, not en masse as it was very friable, containing the product of conception which was imbedded in clot, the blood being in a state of molecular disintegration. It was impossible to distinguish tube and ovary from parts of mass, the layers of external portion of tumor were decolorised and of a pale appearance, showing evidently that there had been several attacks of bleeding. After sac and contents were removed the abdominal cavity was flushed with a normal salt solution; right tube and ovary in healthy condition. One point of rupture was easily seen on anterior surface; wound closed with silkworm-gut; sutures removed on the twelfth day, union complete. Patient made a perfect recovery. The standard surgical technique was observed throughout the operation.

---

The Surgeon-General of the Marine Hospital Service is preparing a translation of the Bertillon classification of the causes of death, to be sent to the various health-officers of this country. It would be a most excellent reform to have this classification generally adopted. Comparison of statistics would be greatly simplified. It is hoped this city will be one of the first to adopt the new plan.—*Cleveland Journal of Medicine.*

